# Differential scanning calorimetry of plasma in glioblastoma: toward a new prognostic / monitoring tool

**DOI:** 10.18632/oncotarget.24317

**Published:** 2018-01-25

**Authors:** Philipp O. Tsvetkov, Emeline Tabouret, Andrei Y. Roman, Sylvie Romain, Céline Bequet, Olga Ishimbaeva, Stéphane Honoré, Dominique Figarella-Branger, Olivier Chinot, François Devred

**Affiliations:** ^1^ Aix-Marseille Université, CNRS, INP, Inst Neurophysiopathol, Faculté de Pharmacie de Marseille, 13385 Marseille, France; ^2^ AP-HM, Hôpital de la Timone, Service de Neuro-Oncologie, 13005 Marseille, France; ^3^ AP-HM, Faculté de Médecine Nord, Service de Transfert d’Oncologie Biologique, 13015 Marseille, France; ^4^ AP-HM, Hôpital de la Timone, Service d'Anatomopathologie, 13005 Marseille, France; ^5^ Institute of Physiologically Active Compounds, RAS, 142432 Chernogolovka, Russian Federation

**Keywords:** glioblastoma, differential scanning calorimetry, disease monitoring, plasma, calorimetric signature

## Abstract

Glioblastoma is the most frequent and aggressive primary brain tumor in adults. Recently, a growing number of studies have shown that denaturation profile of plasma samples obtained by differential scanning calorimetry (DSC) can represent a signature of a disease. In this study, we analyzed for the first time the DSC denaturation profiles of the plasma from patients with recurrent glioblastoma (n=17). Comparison to the one of healthy individuals (n=10) and to already described profiles in others cancer showed clear differences suggesting that this DSC profile may constitute a signature of glioblastoma. Parameters extracted from these profiles were used for cluster analysis which revealed the existence of glioblastoma profile subgroups which correlated with prognostic factors. Moreover, we showed that the presence of circulating bevacizumab and carmustine did not alter this calorimetric signature of the disease, indicating that an evolution of the profile could be followed without being masked by ongoing systemic treatment. Thus, our results constitute a very promising proof of principle that a specific calorimetric profile could be detected in the plasma of glioblastoma patients. Moreover, we believe that our findings point to a potential easy-to-use non-invasive monitoring tool for glioblastoma patients.

## INTRODUCTION

Glioblastoma (GB) is the most frequent and aggressive primary brain tumor in adults. It is characterized by cellular atypia, severe necrosis, and high rate of angiogenesis [1]. To date, response after standard first line treatment associating radiotherapy and temozolomide is very heterogeneous, leading to inevitable recurrence [2]. At relapse, few treatment options are available and remain associated with heterogeneous response rates [3]. In this indication, the use of bevacizumab is associated with favorable response rate and improved progression-free survival [4, 5]. In this context of limited systemic therapies, accurate and timely detection of the disease recurrence is crucial in order to optimize the therapeutic options and to improve the patient management. To date, response assessment has been based on neuro-imaging (MRI), steroid dose and clinical examination, which can be difficult to interpret, especially following radiotherapy, anti-angiogenic therapy or immunotherapy [6, 7]. Thus, the identification of a new easy-to-use method to monitor glioblastoma evolution is an urgent need in the neuro-oncology field.

Recently, it was suggested that differential scanning calorimetry (DSC), a biophysical method routinely used to measure temperature-induced denaturation of purified proteins [8, 9], could have a new disease-monitoring application. For purified single-domain proteins, the profile of denaturation obtained by DSC is usually characterized by a single peak with its surface corresponding to the enthalpy of protein denaturation (ΔH), that is additional energy that should be applied to the protein solution to reach full protein unfolding [10]. The maximum of this peak corresponds to the melting temperature (Tm). These two parameters are characteristic for each protein. They can be altered by protein modification, such as mutations or post-translational modifications [8], or by interaction with its binding partners, such as other proteins, small molecules and even ions [9–11]. It has been recently shown that applying DSC directly to biofluids, such as serum, plasma or cerebro-spinal fluid (CSF), resulted in reproducible specific signature profiles characteristic of the clinical state of an individual [12]. With such complex biological samples, the obtained denaturation profile corresponds to the superposition of denaturation calorimetric profiles of all components in the sample. The modifications in blood composition of patients suffering from cancer can lead to significant changes in calorimetric profile of plasma. Over the past five years, an increasing number of publications have shown that such impact exists in many diseases including diabetes, Lyme disease, as well as several types of cancer [12]. For example, calorimetric profile of plasma samples from patients affected by cervical, endometrial, ovarian or lung cancers, was different from that of healthy controls [13, 14]. In the later studies, the difference between profile of healthy controls and patients has also been observed for breast, multiple myeloma, colorectal and gastric cancer [15–17]. Moreover, superposition of DSC profiles obtained from these cancers suggested that each type of cancer may have a characteristic thermogram [14]. Finally, a preliminary study conducted on the CSF of six GB patients revealed a specific profile suggesting the existence of a biofluid signature of GB [18].

Recurrent GB is generally associated with a certain tumor burden, increasing the chance to detect potential circulating markers. Our primary objective was to analyze the DSC profile of plasma of recurrent GB patients. Our secondary objectives were (i) to correlate this profile to patient characteristics, (ii) to evaluate its potential prognostic value and (iii) to analyze the impact of circulating bevacizumab and carmustine agents on DSC profile.

## RESULTS

### Patient characteristics (Table 1)

**Table 1:** Patient characteristics

Characteristics	N	*%*
**Gender**		
Women	5	*30*
Men	12	*70*
**Type of surgery**		
Gross total resection	10	*59*
Partial resection	7	*41*
**Histology**		
*IDHwt* glioblastoma	17	*100*
**MGMT promoter**		
Methylated	1	*11*
Unmethylated	8	*89*
**First line treatment**		
Radiotherapy alone	1	*6*
Temozolomide alone	1	*6*
Stupp protocol	15	*88*
**Before bevacizumab + BCNU treatment**		
Age (*years*: median, range)	62,5 (42,5-89,8)	
Body Mass Index (median, range)	24,1 (18,1-32,1)	
Delay between initial diagnosis and relapse (*months*: median, range)	13,3 (4,1-64,7)	
Karnofsky Performans Status (median)	70	
60	3	*19*
70	6	*37*
80	3	*19*
90	4	*25*
Patients under steroid	8	*47*
Tumor diameter^*^ (*mm*: median, range)	45 (20-80)	

Wt : wild-type

^*^ Diameter of contrast enhancement

Patient cohort was composed of 17 GB patients with a median age of 62.5 years (range, 42-89) and a median Karnofsky Performance Status (KPS) of 70 (range, 60-90) at relapse. For the majority of patients, the first line treatment consisted of surgical resection followed by radio-chemotherapy. At relapse, median diameter of enhanced lesion(s) was 45mm (range, 20-80). The control cohort used in this study was composed of 2 women and, 8 men, with a median age of 36.7 years (range, 24-51) and a body mass index of 23.3 (range, 19.2-30).

### DSC profiling of plasma samples

**Figure 1:**
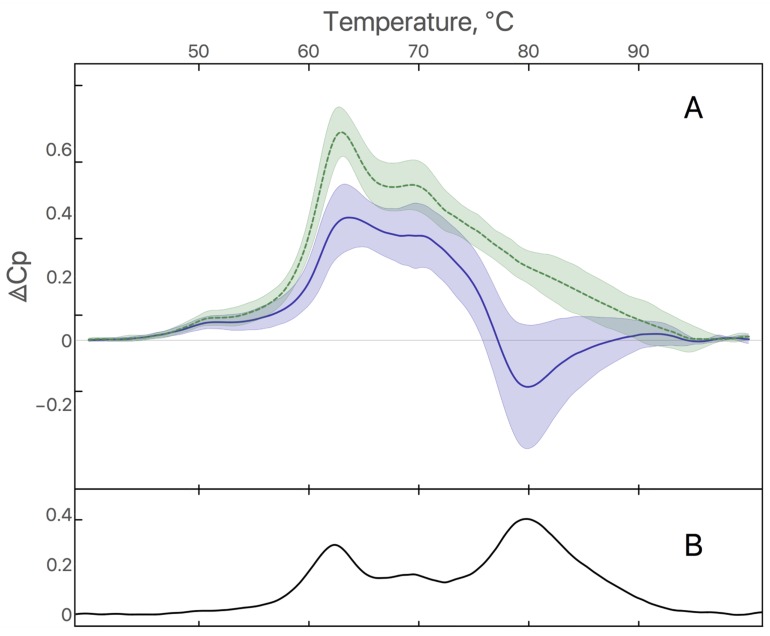
**(A)** Average of plasma denaturation profiles from 10 healthy individuals (green dashed curve) and 17 GB patients (blue solid curve). Filled area corresponds to standard deviation. **(B)** Difference between average of plasma denaturation profiles from healthy individuals and GB patients.

Plasma samples from 10 healthy individuals and 17 GB patients were analyzed by DSC to obtain denaturation profiles. Average profiles of healthy individuals and GB patients are shown in the Figure 1A. Plasma denaturation profiles of healthy individuals are homogeneous and are characterized by a main maximum at 63°C and smaller one at 70°C as previously described [12]. Profiles of GB patients are characterized by a negative peak around 80°C and a less pronounced difference between maxima at 63°C and 70°C. The difference between healthy individuals and GB profiles is significant in the temperature ranges 60-65°C and 75-85°C (p<0.001) (Figure 1B).

### Correlation of DSC parameters with patient characteristics and survival

**Figure 2:**
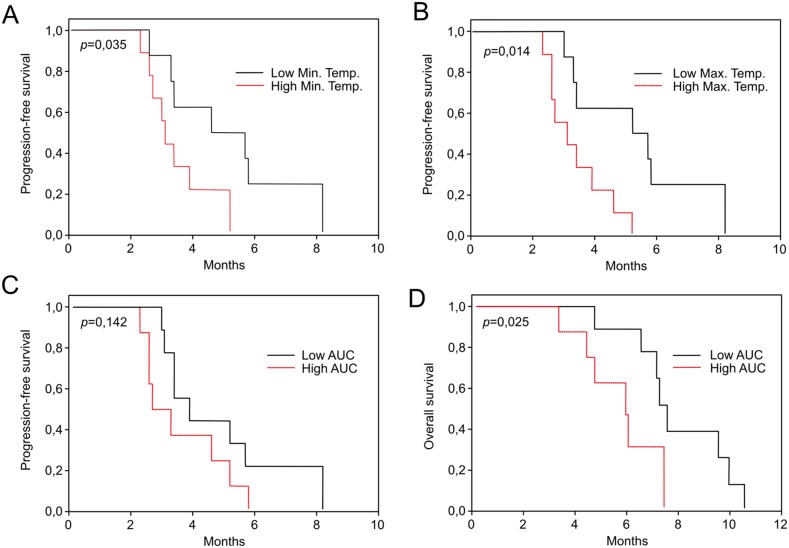
Progression-free survival according to the temperature of minimal **(A)** and maximal **(B)** values of denaturation profiles of GB patients, dichotomized by the median value. Progression-Free survival **(C)** and overall survival **(D)** according the Area Under the Curve (AUC) values of denaturation profiles of GB patients, dichotomized by the median value.

In order to evaluate the potential correlation between patient characteristics and DSC profile, minimal and maximal curve values, associated temperatures, and areas under the curve were extracted from the individual profiles obtained from GB patients. None of these parameters were correlated to classical patient prognostic factors (age, steroid dose, KPS) or other patient characteristics (gender, body mass index). Moreover, DSC features were not correlated to the neuro-imaging characteristics of patients (maximal diameter of enhanced lesion) or to the trough concentration of bevacizumab at D21. Remarkably, several of DSC parameters turned out to have a prognostic value. Indeed, the temperatures of minimal (*p*=0.035) and maximal (*p*=0.014) curve values were associated with progression-free survival (Figure 2A and 2B), while the area under the curve was significantly associated with overall survival (*p*=0.025) (Figure 2C and 2D).

**Figure 3:**
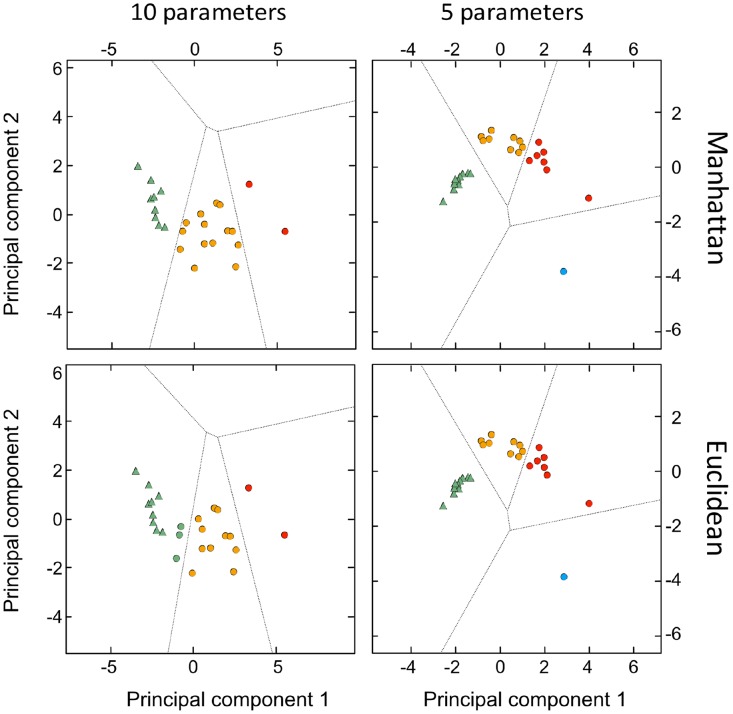
Principal component analysis (PCA)-based clustering by local optimization The two major principal components explain 88% of the total variance plotted for the 10 healthy individuals and 17 GB patients. The different groups are indicated by different colors (triangles for healthy individuals and circles for GB patients).

Despite of our limited number of samples, in an exploratory perspective, we conducted a cluster analysis in order to take into account more DSC profile parameters (see Methods) in a comprehensive way and to perform a finer statistical analysis of the thermograms. We have applied principal components analysis followed by several clustering methods and distance functions most of which clearly separated healthy from GB profiles. For example, as shown in Figure 3, analysis using “Optimize” method with Euclidian distance function showed the existence of two distinct groups of signatures (p<0.005) corresponding to healthy controls (triangles) and GB patients (circles). Moreover, using this method we were also able to identify 3 clusters within the GB group (different colors) which were significantly different from each other in terms of Progression Free Survival (*p*<0.005) and overall survival (*p*=0.03). Using Manhattan distance function gave identical clusters.

Finally, we analyzed the association between clusters or DSC features with the time between the end of radiotherapy and relapse and found no correlation.

### Impact of treatment on DSC profile

**Figure 4:**
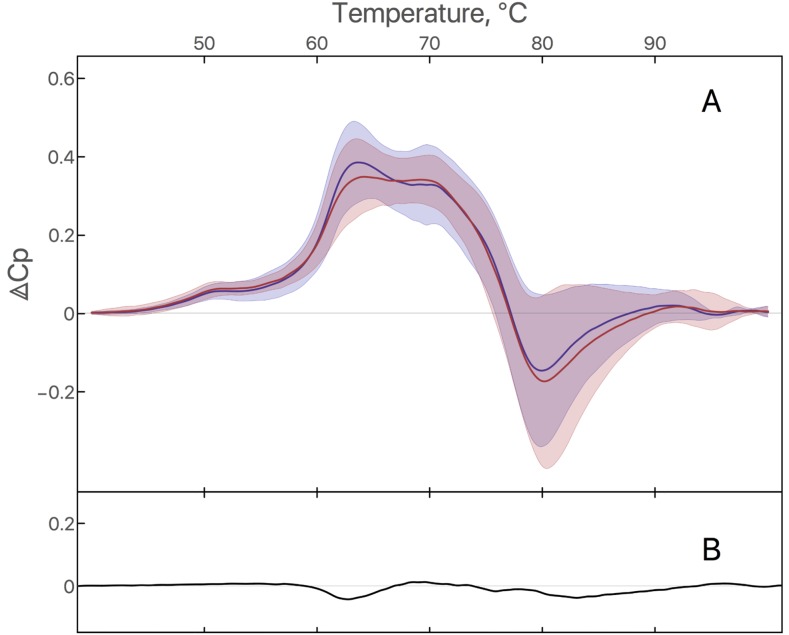
**(A)** DSC signature of plasma from GB patients at D1 and D21: Average of plasma denaturation profiles from 17 GB patients at D1 (blue curve) and the same 17 GB patients at D21 (red curve). Filled area corresponds to standard deviation. **(B)** Difference between average of plasma denaturation profiles of GB patients at D21 and D1.

To test the impact of a systemic pharmacologic treatment on the denaturation profile, we compared plasma samples before and 21 days after treatment with bevacizumab and carmustine (Figure 4A). No significant modifications of DSC parameters were observed between the first (D1) and the second calorimetric profile (D21), suggesting that remaining circulating bevacizumab and carmustine in plasma do not interfere with the GB calorimetric profile (Figure 4B). When the patients were stratified according to the objective response (responders *versus* non responders), no significant difference was observed at D21 in the neither subgroups.

## DISCUSSION

DSC has been previously used to detect specific biofluid denaturation profiles in autoimmune and infectious diseases, chronic health conditions as well as in a growing range of cancers [12]. In this report, we show for the first time the existence of a specific plasma denaturation profile of patients with recurrent GB which differs from the profile of healthy individuals and from all previously published disease profiles. The presence of such profile in plasma of GB patients thus opens up the path for a potential new method for monitoring of GB. DSC profiles were previously analyzed in the cerebrospinal fluid of six GB patients showing interesting but limited results [18]. In another study, this technique has been applied directly to the brain tissue showing differences between distinct stages of gliomas as well as control brain tissue [19]. However, this technique is limited by the amount of tissue required and by the access to the tumor. In our study, such a signature was observed in plasma samples. Since drawing blood is less invasive compared to lumbar puncture, this method would be much easier to implement for daily practice in clinics, making it an effective and convenient tool for disease monitoring. Indeed, sample analysis by DSC is a well-established method that is highly reproducible, relatively inexpensive, demands simple processing and requires little plasma material. Thus, plasma denaturation signatures could represent a valuable addition to the current tools that monitor the disease and guide clinical GB management.

In our study, we have also shown that these profiles can be analyzed automatically using classification methods and that this classification unambiguously separated profiles between healthy and GB. In an exploratory approach, we also found clustering settings that revealed subgroups in the GB population with different prognostic values. The DSC profile appears to be related to prognosis independently of the classical patient prognostic factors, such as age and KPS or other patient characteristics such as gender, body mass index. This prognostic value might be helpful during the course of the disease, although considering our small number of samples it will need to be confirmed in a larger cohort and to be tested also in the setting of the initial GB diagnosis. Interestingly, we did not observe significant differences between profiles obtained before and after bevacizumab treatment. Since second plasma sample was collected only three weeks after the beginning of treatment, it was probably too early to observe any correlation between an evolution of the DSC profile and disease response. Nevertheless in this context, our results suggest that the presence of circulating drugs, such as bevacizumab, does not alter the calorimetric signature of the disease, indicating that an evolution of the profile could be followed without being masked by ongoing treatment. For all these reasons, our findings constitute a promising proof of principle that DSC could be used for a better management of GB patients.

In addition to the potential clinical application of DSC, it can also be used to improve our understanding of the mechanism of GB development. Indeed, DSC denaturation profile of a complex solution such as plasma corresponds to superposition of denaturation calorimetric profiles of all components in the plasma. In practice, the overall profile corresponds to the denaturation of the most abundant proteins, which are albumin, immunoglobulin G, fibrinogen, immunoglobuline A, alpha2macroglobulin, haptoglobulin and alpha1antitrypsin [12, 14]. Blood homeostasis maintains the concentration of these proteins constant in any healthy organism, resulting in a reproducible “healthy” denaturation signature. A modification of the profile is a sign of a change in the homeostasis which can have several causes: a change in the concentration of the most abundant proteins, any post-translation chemical modification of one of them or the binding of a ligand, constituting a potential biomarker. Thus, this method could reveal the presence of biomarkers in the plasma directly by their impact on the calorimetric profile.

Despite several limitations, such as the limited number of samples and the monocentric nature of our study, we believe that this study constitutes the proof of principle that DSC could be used as a new approach for prognostic evaluation and/or disease monitoring with minimal handling of plasma samples. Even though our results are statistically significant, they should be confirmed in a larger population to evaluate the robustness of our approach. It would also be interesting to analyze the DSC profile of patients’ plasma sample at initial diagnosis and during the treatment in a larger prospective cohort, including other forms of gliomas. It will help to identify potential specific variations of DSC profile according to grading, prognostic and/or treatment, and thus address the ability of DSC as a new marker of glioblastoma.

## MATERIALS AND METHODS

### Patients

We retrospectively included all adult patients referred to our institution for recurrent *IDH* wild-type glioblastoma who received bevacizumab at the dose of 15 mg/kg every 3 weeks in association with carmustine at the dose of 150 mg/m^2^ every 6 weeks for whom plasma samples were available (Assistance Publique-Hôpitaux de Marseille Tumor Bank, authorization number 2013–1786) before bevacizumab administration (D1) and at the time of the second bevacizumab administration (D21). Clinical and imaging evaluations were performed every 3 weeks and 6 weeks respectively. Treatment responses and disease progression were reviewed using the RANO criteria [20]. All patients provided written informed consent in accordance with institutional, national guidelines and the Declaration of Helsinki.

### Plasma samples

Plasma samples were collected at baseline, before bevacizumab administration (D1), and before the second bevacizumab administration (D21). Clinical data and neuro-imaging characteristics (when available) were recorded and the bevacizumab trough concentration was determined before the second administration. Plasmas from 10 healthy controls were also collected and processed in an identical way to the patients’ samples (blood drawn in the hospital, plasma stored in the same biobank) before being analyzed. Plasma concentration of bevacizumab was quantified by enzyme linked immunosorbent assay (Elisa, Theradiag, Croissy Beaubourg, France) [21].

### Sample preparation

Unprocessed plasma samples were stored at −80°C. No other specific purification step was added in order not to perturb the interactome or alter the chemical state of plasma proteins. Before DSC analysis, samples were thawed rapidly at 37°C and then diluted 25 times into the final volume of 1 mL of phosphate-buffered saline (PBS) buffer with sodium citrate 13 mM. Diluted samples were stored at −20°C when not in use.

### Sample analysis by DSC

DSC thermograms were collected using a high-sensitivity differential scanning VP-DSC microcalorimeter (MicroCal, Northampton, MA, now part of Malvern Instruments Ltd) according to the manufacturer’s instructions. 580 μL of sample were used to fill the DSC chamber. Scans were recorded from 10°C to 110°C with a ramp heating rate of 1°C/min using the midfeedback mode and a filtering period of 2s. Thermograms were treated using software Origin 7 (OriginLab Corporation, Northampton, MA) as previously described [22]. After the first run was completed, a second run of the same sample was performed without emptying the cell and subtracted from the raw data obtained in the first scan. Thermograms were plotted as the excess partial molar heat capacity (cal K^-1^ mol^-1^) versus temperature (°C). Global protein concentration was not normalized so that the profiles obtained reflected the possible differences in protein content of plasma.

### Statistical analyses

Categorical variables were presented as frequencies and percentages, continuous variables as median and range. For survival analyses, continuous variables were dichotomized by the median value. Overall survival (OS) was defined to be the time from first bevacizumab administration to death from any cause, censored at the date of last contact. Progression-Free Survival (PFS) was the time from first bevacizumab administration to documented progression or death, censored at the date of the last documented disease evaluation. Kaplan-Meier method was used to estimate survivals distributions. Log-rank tests were used for univariate comparisons. Mann-Whitney U-test was used to compare quantitative and qualitative values; qualitative values were analyzed by Fisher exact test and Chi 2 test. Correlations were analyzed using the Spearman test. All reported *p*-values were two-sided, and *p<*0.05 was considered statistically significant. Minimal and maximal curve values, associated temperatures, and areas under the curve were then extracted from the denaturation profiles to be compared directly between patients and controls. All statistical analyses were performed by PASW Statistics 22 from IBM Corporation.

To explore the DSC profiles by principal component and clustering analysis, we used either 10 or the 5 following profile parameters: heat capacity (cal K^−1^ mol^−1^) and temperature (°C) of the negative peak; the minimal and the maximal values of the heat capacity as well as the temperature where the heat capacity is minimal. Standardization (z-score) and cluster analysis were performed in Mathematica 10 software from Wolfram Research using the following methods: ”Agglomerate” which finds clustering hierarchically, “Optimize” which finds clustering by local optimization, “KMeans” which uses k-means clustering algorithm, “KMedoids” which partitions around medoids and “Spectral” which uses spectral clustering algorithm, with Manhattan or Euclidian distances.

## CONCLUSION

In conclusion, we describe a novel method which presents the advantage of revealing the presence of markers in the plasma directly by their impact on the DSC denaturation profile. This approach not only opens the path for rational search of new biomarkers, but also more importantly opens up the perspective of a new easy-to-use non-invasive monitoring tool for GB patients. A larger study will now be needed to confirm the specificity of the signature and the extent of its prognostic value and the way its profile changes during treatment.
